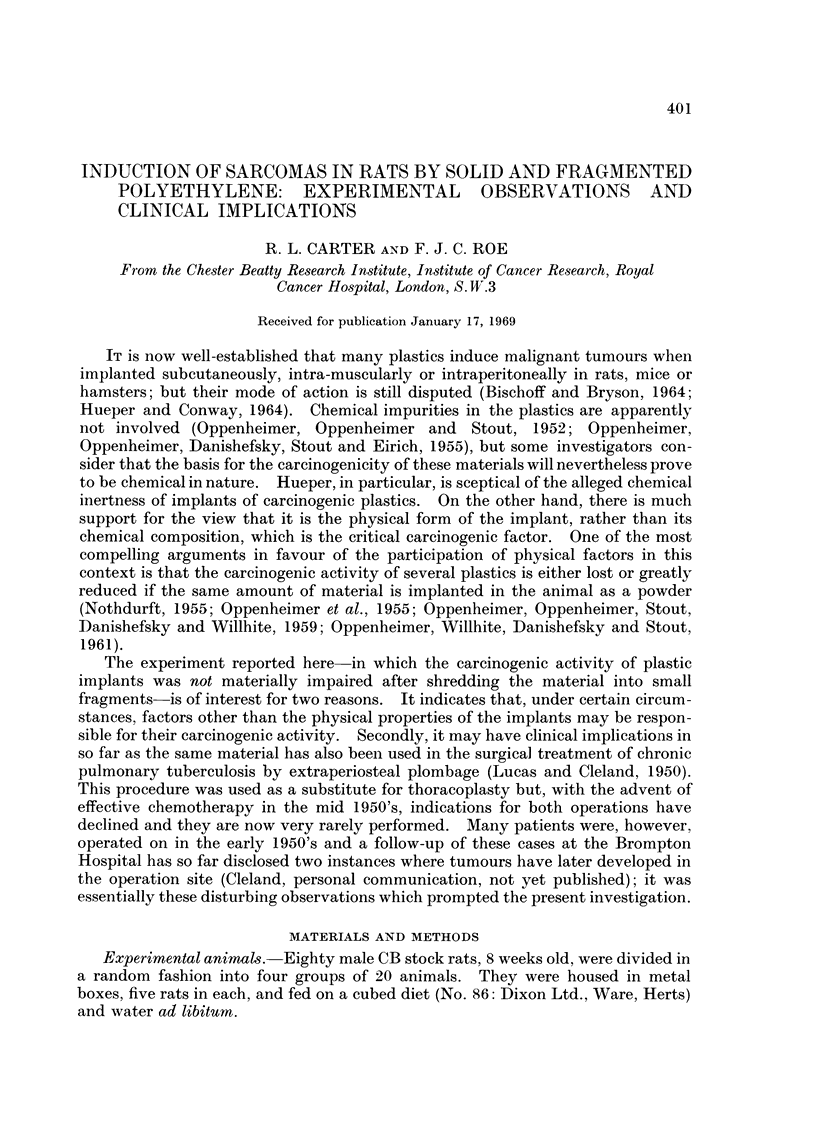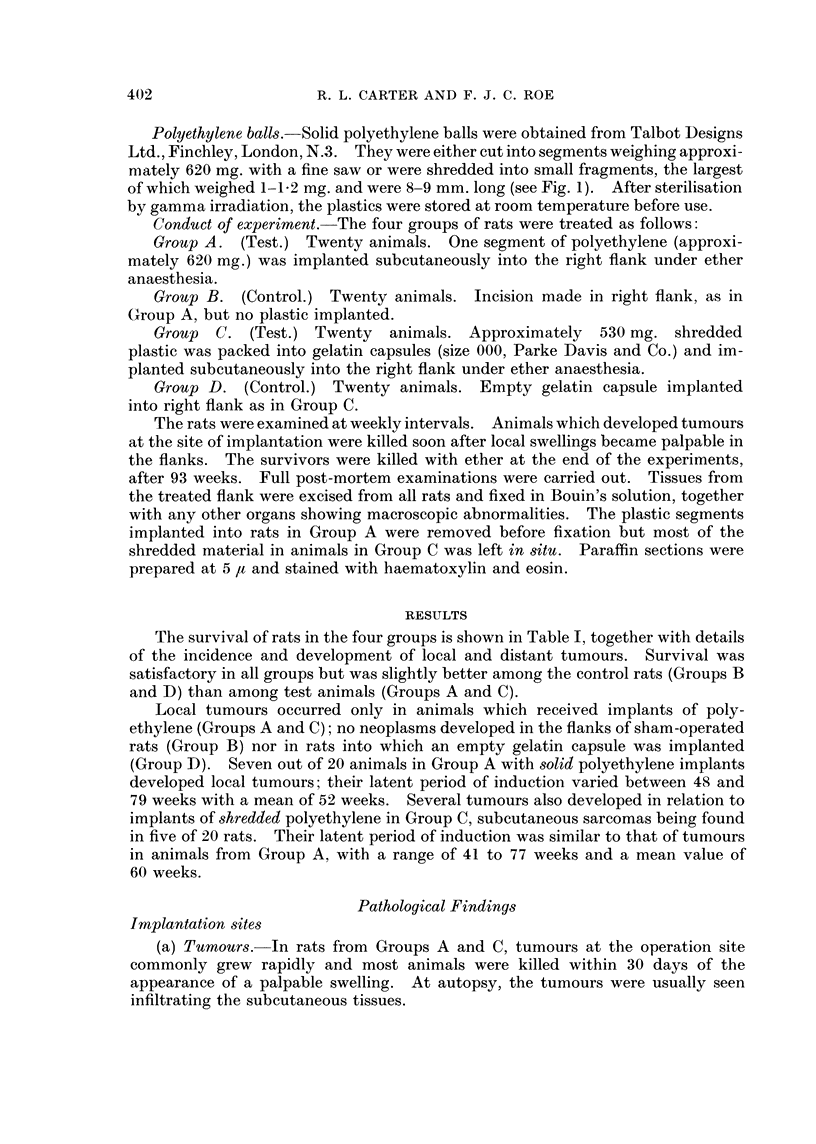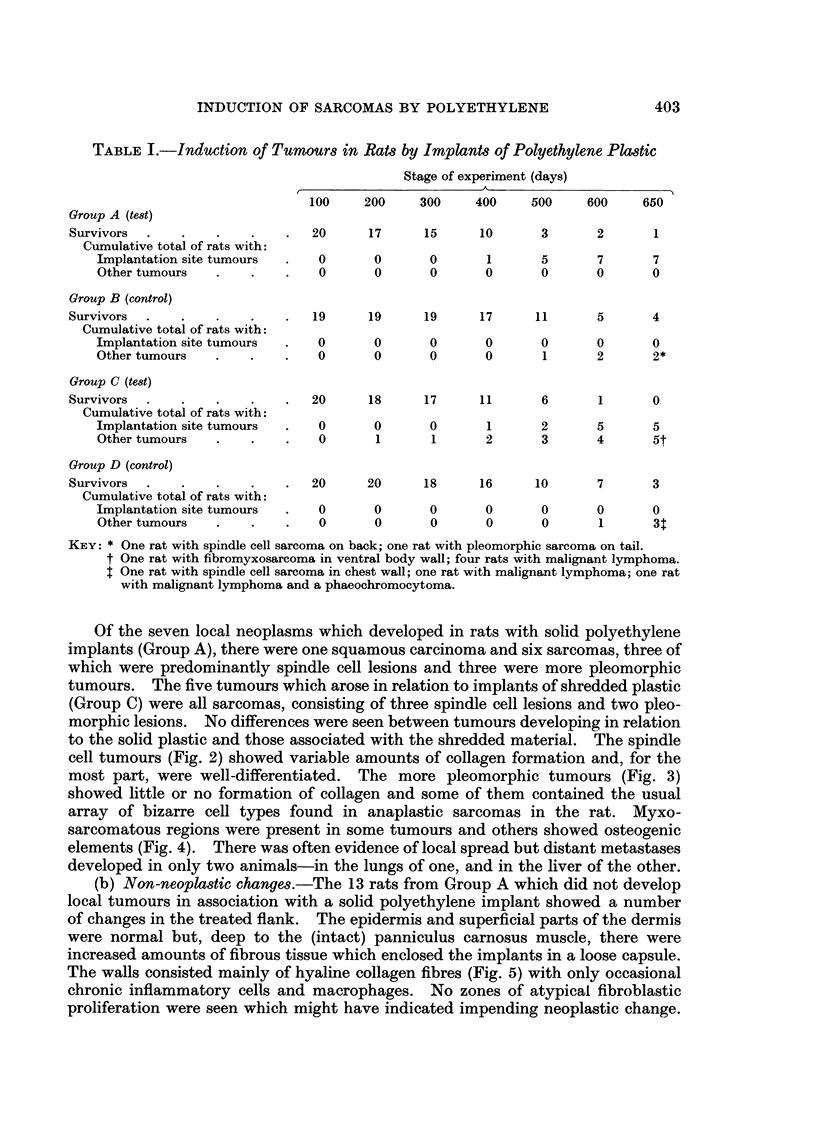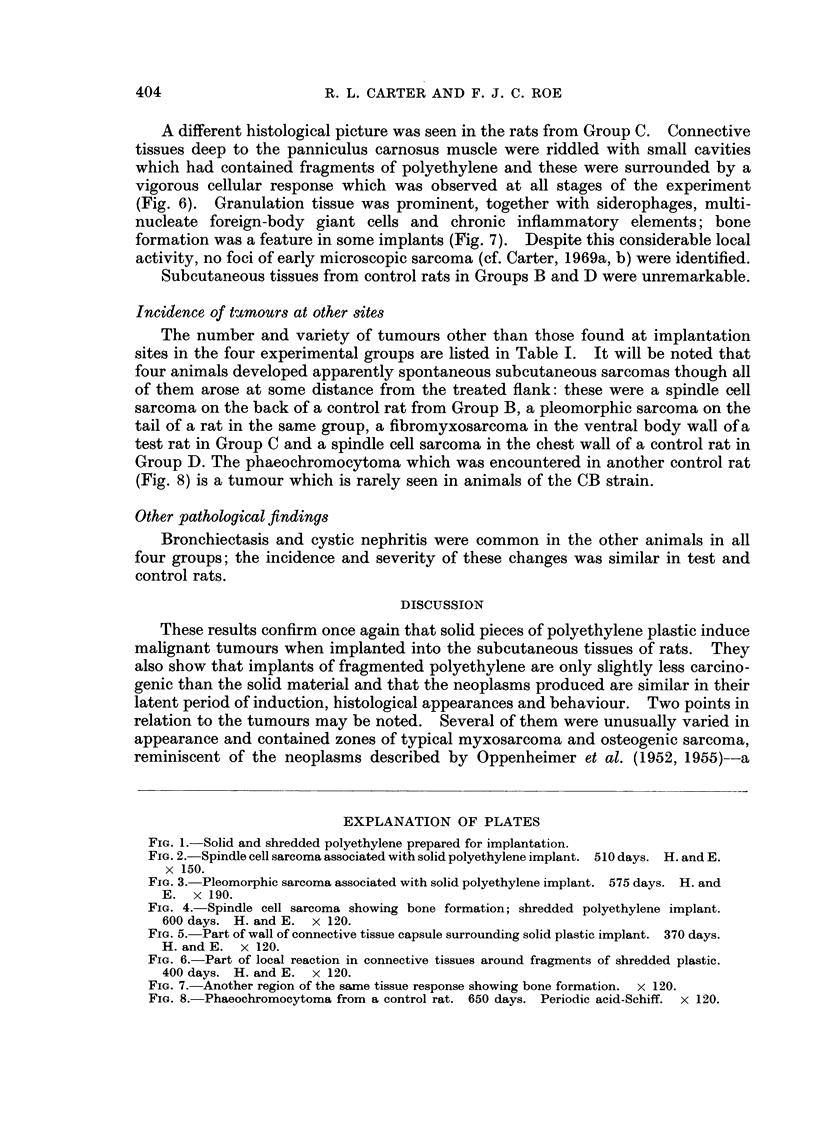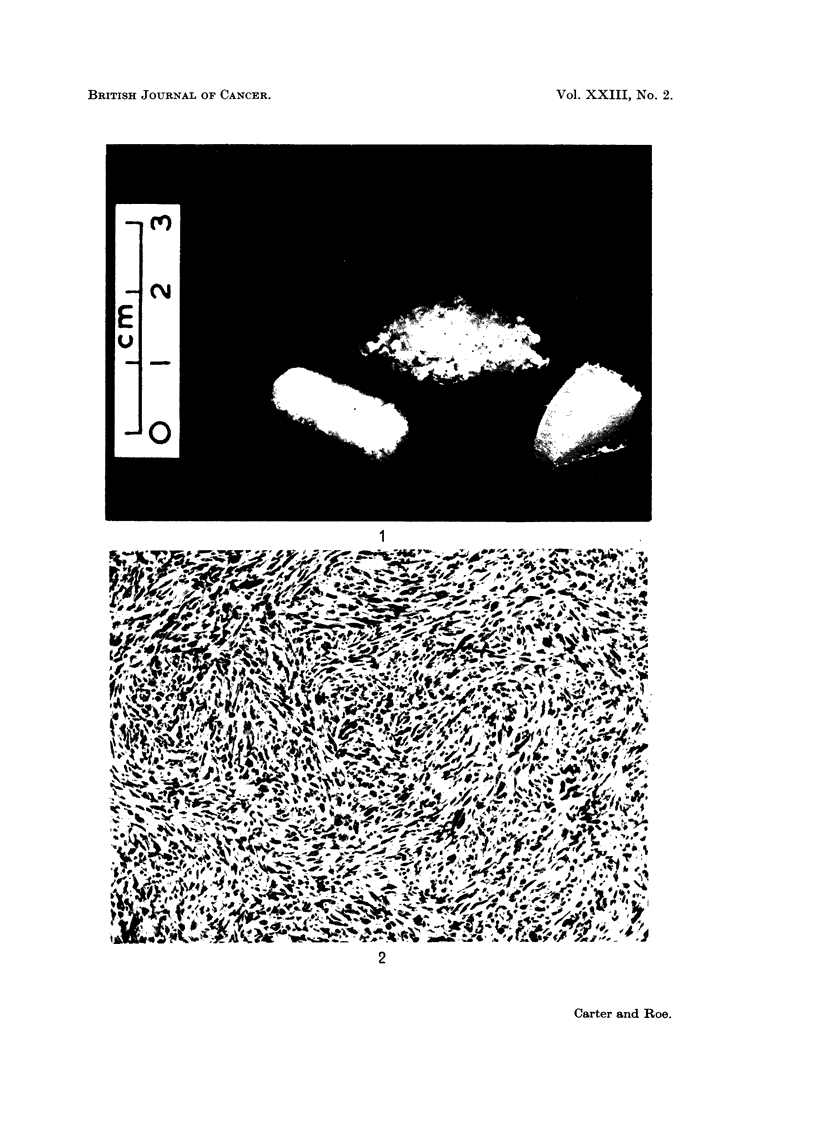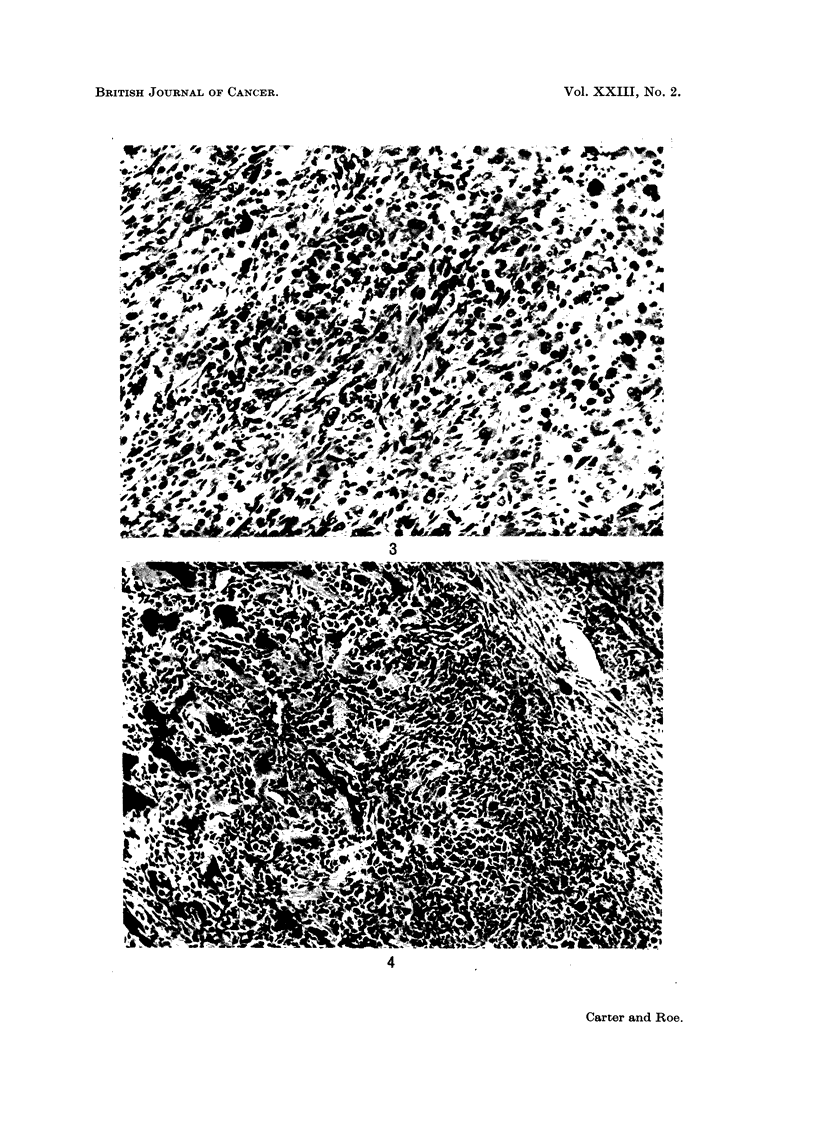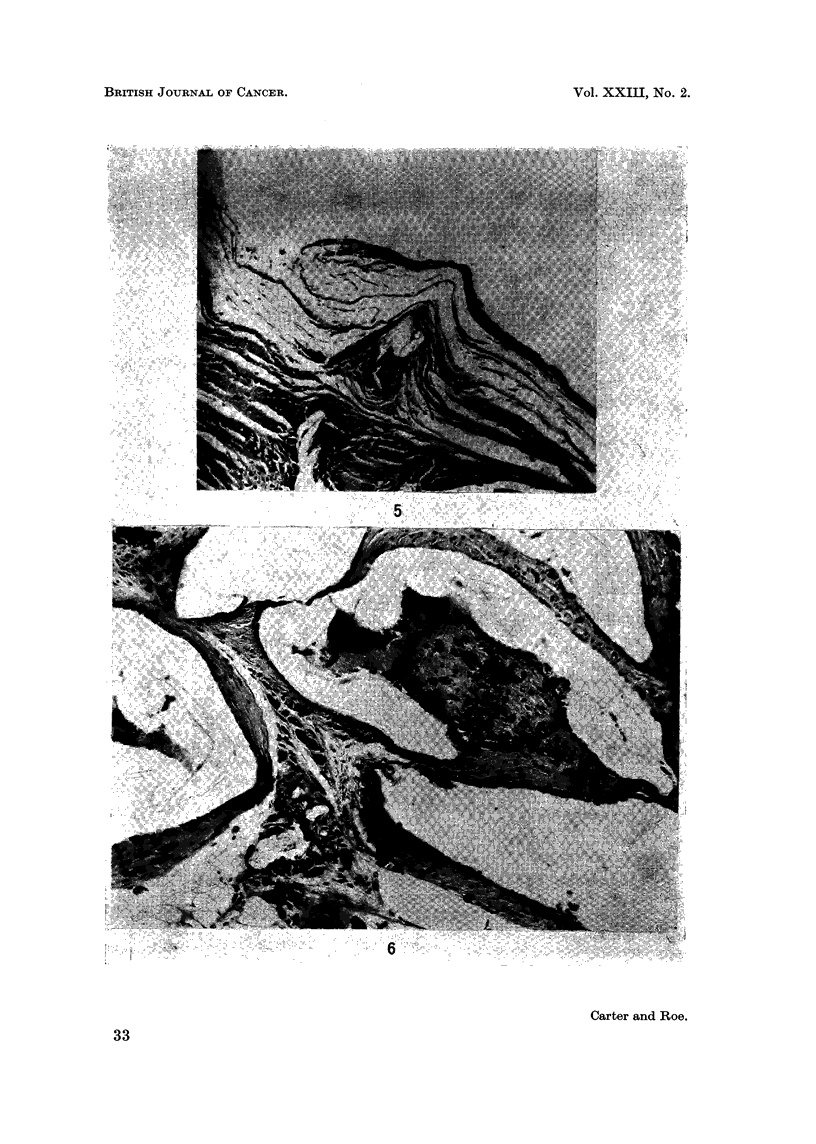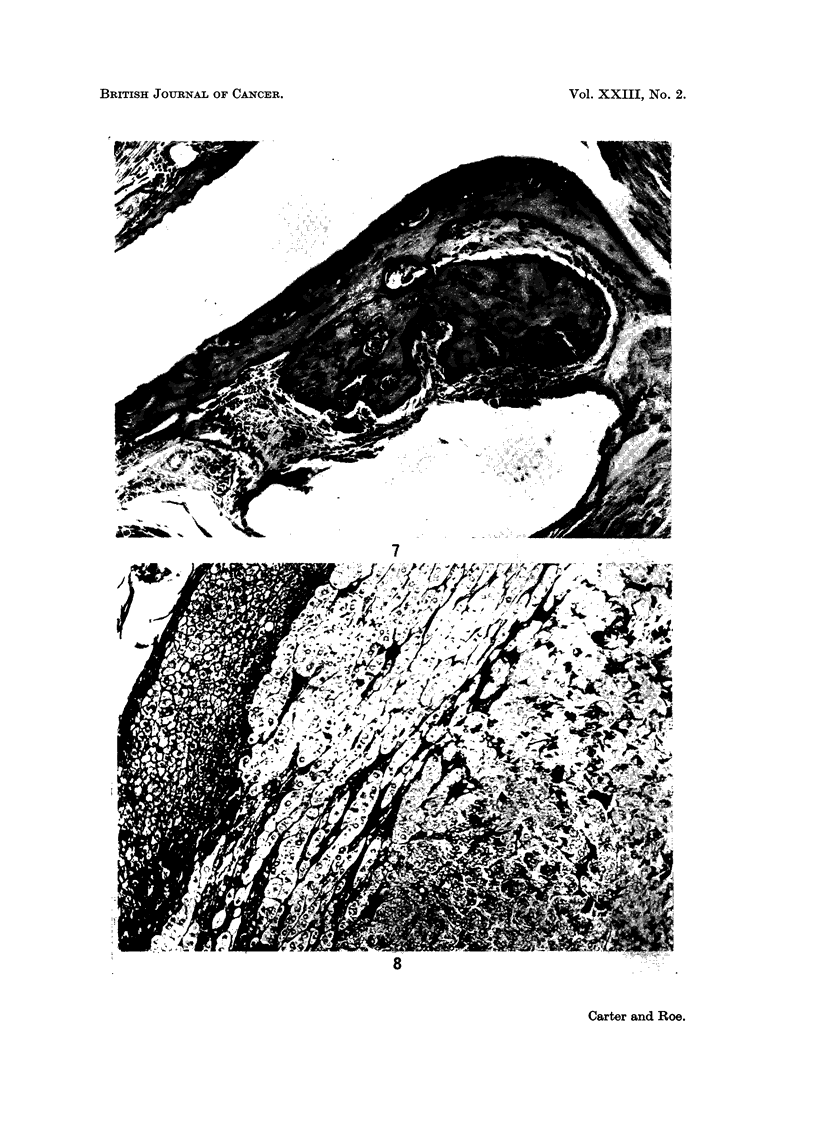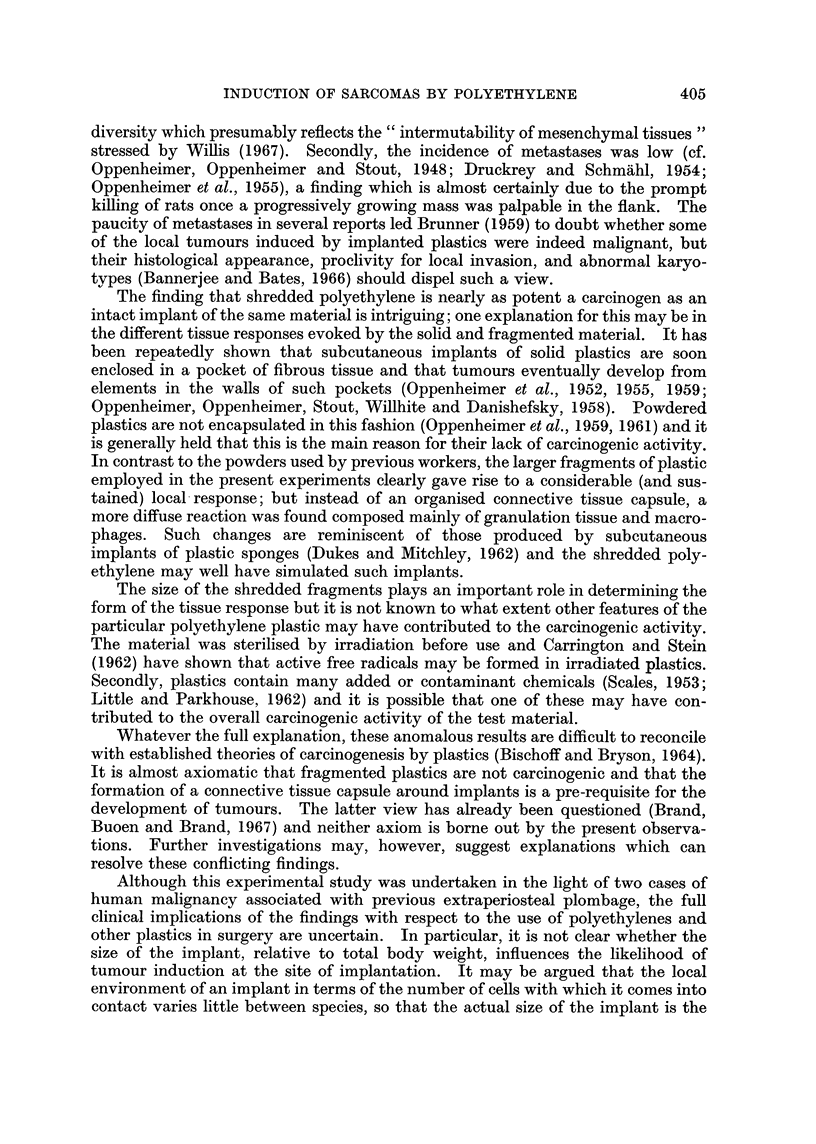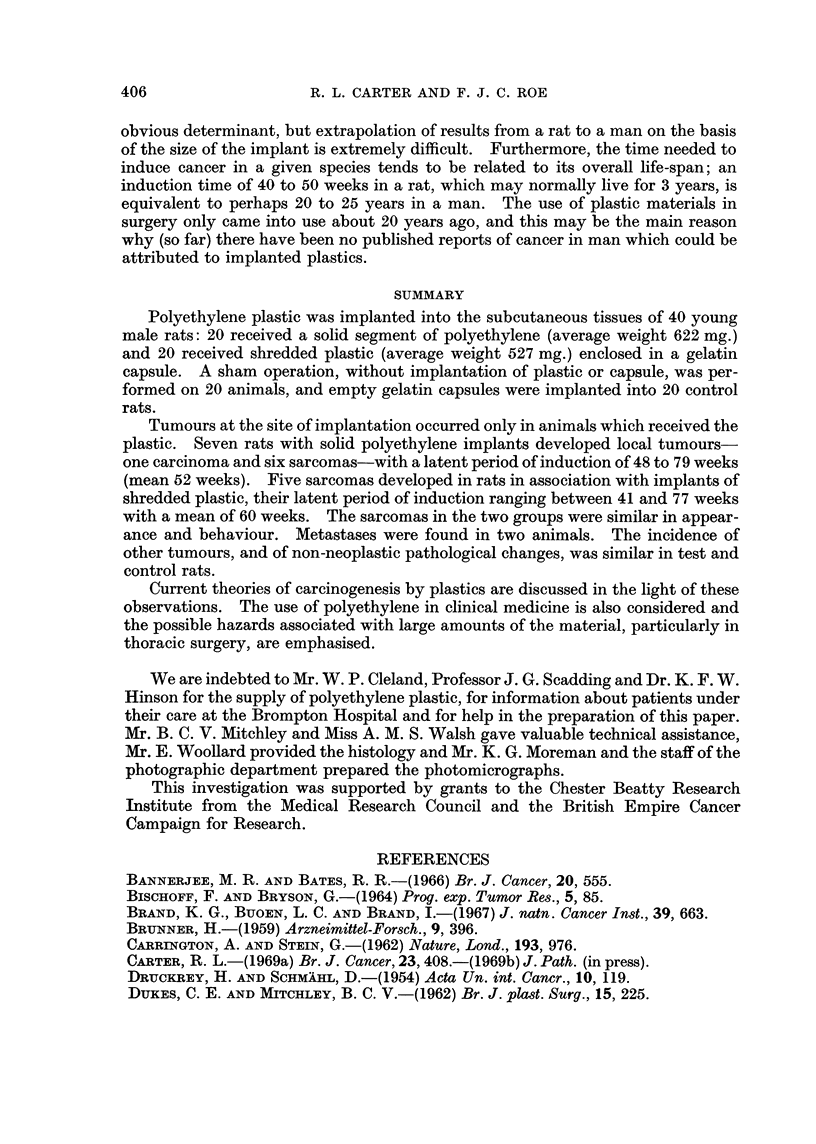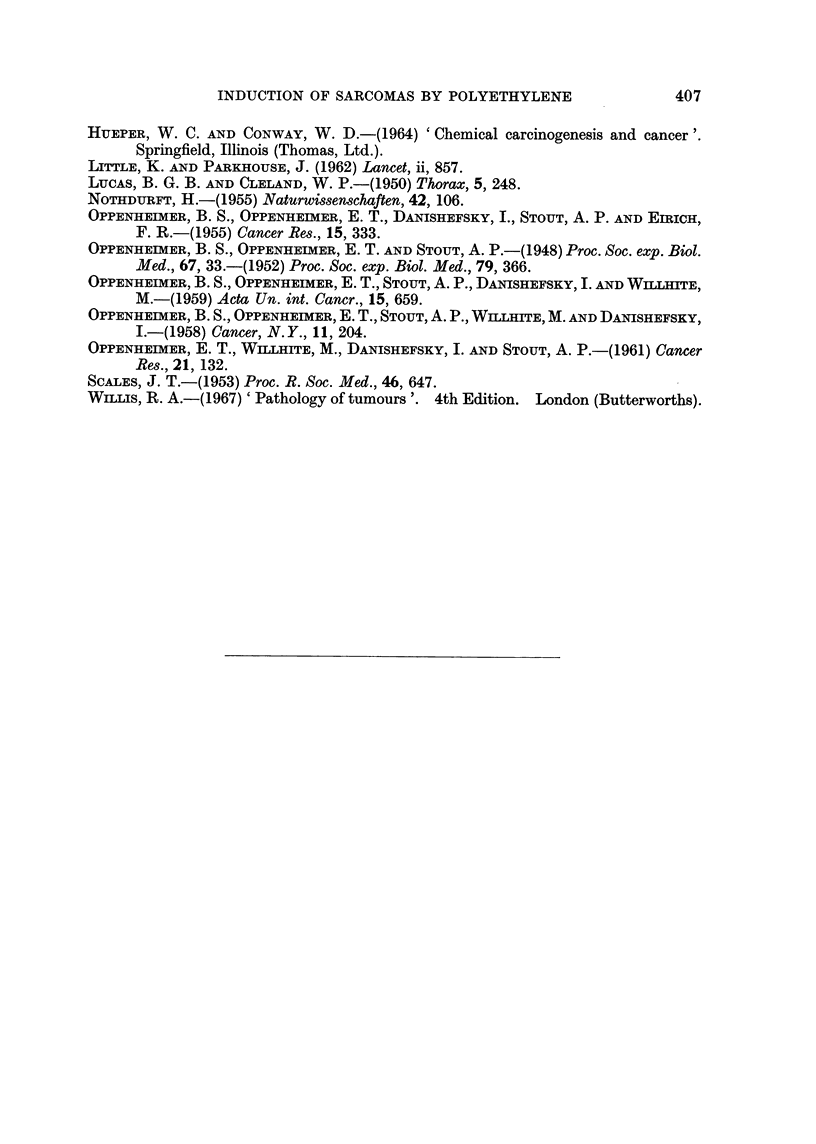# Induction of sarcomas in rats by solid and fragmented polyethylene: experimental observations and clinical implications.

**DOI:** 10.1038/bjc.1969.52

**Published:** 1969-06

**Authors:** R. L. Carter, F. J. Roe

## Abstract

**Images:**


### INDUCTION OF SARCOMAS IN RATS BY SOLID AND FRAGMENTED POLYETHYLENE: EXPERIMENTAL OBSERVATIONS AND CLINICAL IMPLICATIONS

R. L. CARTER AND F. J. C. ROE

*From the Chester Beatty Research Institute, Institute of Cancer Research, Royal Cancer Hospital, London, S.W.3*

Received for publication January 17, 1969

It is now well-established that many plastics induce malignant tumours when implanted subcutaneously, intra-muscularly or intraperitoneally in rats, mice or hamsters; but their mode of action is still disputed (Bischoff and Bryson, 1964; Hueper and Conway, 1964). Chemical impurities in the plastics are apparently not involved (Oppenheimer, Oppenheimer and Stout, 1952; Oppenheimer, Oppenheimer, Danishefsky, Stout and Eirich, 1955), but some investigators consider that the basis for the carcinogenicity of these materials will nevertheless prove to be chemical in nature. Hueper, in particular, is sceptical of the alleged chemical inertness of implants of carcinogenic plastics. On the other hand, there is much support for the view that it is the physical form of the implant, rather than its chemical composition, which is the critical carcinogenic factor. One of the most compelling arguments in favour of the participation of physical factors in this context is that the carcinogenic activity of several plastics is either lost or greatly reduced if the same amount of material is implanted in the animal as a powder (Nothdurft, 1955; Oppenheimer *et al.*, 1955; Oppenheimer, Oppenheimer, Stout, Danishefsky and Willhite, 1959; Oppenheimer, Willhite, Danishefsky and Stout, 1961).

The experiment reported here—in which the carcinogenic activity of plastic implants was *not* materially impaired after shredding the material into small fragments—is of interest for two reasons. It indicates that, under certain circumstances, factors other than the physical properties of the implants may be responsible for their carcinogenic activity. Secondly, it may have clinical implications in so far as the same material has also been used in the surgical treatment of chronic pulmonary tuberculosis by extraperiosteal plombage (Lucas and Cleland, 1950). This procedure was used as a substitute for thoracoplasty but, with the advent of effective chemotherapy in the mid 1950's, indications for both operations have declined and they are now very rarely performed. Many patients were, however, operated on in the early 1950's and a follow-up of these cases at the Brompton Hospital has so far disclosed two instances where tumours have later developed in the operation site (Cleland, personal communication, not yet published); it was essentially these disturbing observations which prompted the present investigation.

### MATERIALS AND METHODS

*Experimental animals.*—Eighty male CB stock rats, 8 weeks old, were divided in a random fashion into four groups of 20 animals. They were housed in metal boxes, five rats in each, and fed on a cubed diet (No. 86: Dixon Ltd., Ware, Herts) and water *ad libitum.*

*Polyethylene balls.*—Solid polyethylene balls were obtained from Talbot Designs Ltd., Finchley, London, N.3. They were either cut into segments weighing approximately 620 mg. with a fine saw or were shredded into small fragments, the largest of which weighed 1–1·2 mg. and were 8–9 mm. long (see Fig. 1). After sterilisation by gamma irradiation, the plastics were stored at room temperature before use.

*Conduct of experiment.*—The four groups of rats were treated as follows:

*Group A.* (Test.) Twenty animals. One segment of polyethylene (approximately 620 mg.) was implanted subcutaneously into the right flank under ether anaesthesia.

*Group B.* (Control.) Twenty animals. Incision made in right flank, as in Group A, but no plastic implanted.

*Group C.* (Test.) Twenty animals. Approximately 530 mg. shredded plastic was packed into gelatin capsules (size 000, Parke Davis and Co.) and implanted subcutaneously into the right flank under ether anaesthesia.

*Group D.* (Control.) Twenty animals. Empty gelatin capsule implanted into right flank as in Group C.

The rats were examined at weekly intervals. Animals which developed tumours at the site of implantation were killed soon after local swellings became palpable in the flanks. The survivors were killed with ether at the end of the experiments, after 93 weeks. Full post-mortem examinations were carried out. Tissues from the treated flank were excised from all rats and fixed in Bouin's solution, together with any other organs showing macroscopic abnormalities. The plastic segments implanted into rats in Group A were removed before fixation but most of the shredded material in animals in Group C was left *in situ*. Paraffin sections were prepared at 5 $\mu$ and stained with haematoxylin and eosin.

### RESULTS

The survival of rats in the four groups is shown in Table I, together with details of the incidence and development of local and distant tumours. Survival was satisfactory in all groups but was slightly better among the control rats (Groups B and D) than among test animals (Groups A and C).

Local tumours occurred only in animals which received implants of polyethylene (Groups A and C); no neoplasms developed in the flanks of sham-operated rats (Group B) nor in rats into which an empty gelatin capsule was implanted (Group D). Seven out of 20 animals in Group A with *solid* polyethylene implants developed local tumours; their latent period of induction varied between 48 and 79 weeks with a mean of 52 weeks. Several tumours also developed in relation to implants of *shredded* polyethylene in Group C, subcutaneous sarcomas being found in five of 20 rats. Their latent period of induction was similar to that of tumours in animals from Group A, with a range of 41 to 77 weeks and a mean value of 60 weeks.

### *Pathological Findings*

#### *Implantation sites*

(a) *Tumours.*—In rats from Groups A and C, tumours at the operation site commonly grew rapidly and most animals were killed within 30 days of the appearance of a palpable swelling. At autopsy, the tumours were usually seen infiltrating the subcutaneous tissues.

TABLE I.—*Induction of Tumours in Rats by Implants of Polyethylene Plastic*

| | Stage of experiment (days) | | | | | | |
|---|---|---|---|---|---|---|---|
| | 100 | 200 | 300 | 400 | 500 | 600 | 650 |
| *Group A* (*test*) | | | | | | | |
| Survivors . . . . . . | 20 | 17 | 15 | 10 | 3 | 2 | 1 |
| Cumulative total of rats with: | | | | | | | |
| Implantation site tumours . | 0 | 0 | 0 | 1 | 5 | 7 | 7 |
| Other tumours . . . | 0 | 0 | 0 | 0 | 0 | 0 | 0 |
| *Group B* (*control*) | | | | | | | |
| Survivors . . . . . . | 19 | 19 | 19 | 17 | 11 | 5 | 4 |
| Cumulative total of rats with: | | | | | | | |
| Implantation site tumours . | 0 | 0 | 0 | 0 | 0 | 0 | 0 |
| Other tumours . . . | 0 | 0 | 0 | 0 | 1 | 2 | 2* |
| *Group C* (*test*) | | | | | | | |
| Survivors . . . . . . | 20 | 18 | 17 | 11 | 6 | 1 | 0 |
| Cumulative total of rats with: | | | | | | | |
| Implantation site tumours . | 0 | 0 | 0 | 1 | 2 | 5 | 5 |
| Other tumours . . . | 0 | 1 | 1 | 2 | 3 | 4 | 5† |
| *Group D* (*control*) | | | | | | | |
| Survivors . . . . . . | 20 | 20 | 18 | 16 | 10 | 7 | 3 |
| Cumulative total of rats with: | | | | | | | |
| Implantation site tumours . | 0 | 0 | 0 | 0 | 0 | 0 | 0 |
| Other tumours . . . | 0 | 0 | 0 | 0 | 0 | 1 | 3‡ |

KEY: * One rat with spindle cell sarcoma on back; one rat with pleomorphic sarcoma on tail.
† One rat with fibromyxosarcoma in ventral body wall; four rats with malignant lymphoma.
‡ One rat with spindle cell sarcoma in chest wall; one rat with malignant lymphoma; one rat with malignant lymphoma and a phaeochromocytoma.

Of the seven local neoplasms which developed in rats with solid polyethylene implants (Group A), there were one squamous carcinoma and six sarcomas, three of which were predominantly spindle cell lesions and three were more pleomorphic tumours. The five tumours which arose in relation to implants of shredded plastic (Group C) were all sarcomas, consisting of three spindle cell lesions and two pleomorphic lesions. No differences were seen between tumours developing in relation to the solid plastic and those associated with the shredded material. The spindle cell tumours (Fig. 2) showed variable amounts of collagen formation and, for the most part, were well-differentiated. The more pleomorphic tumours (Fig. 3) showed little or no formation of collagen and some of them contained the usual array of bizarre cell types found in anaplastic sarcomas in the rat. Myxosarcomatous regions were present in some tumours and others showed osteogenic elements (Fig. 4). There was often evidence of local spread but distant metastases developed in only two animals—in the lungs of one, and in the liver of the other.

(b) *Non-neoplastic changes.*—The 13 rats from Group A which did not develop local tumours in association with a solid polyethylene implant showed a number of changes in the treated flank. The epidermis and superficial parts of the dermis were normal but, deep to the (intact) panniculus carnosus muscle, there were increased amounts of fibrous tissue which enclosed the implants in a loose capsule. The walls consisted mainly of hyaline collagen fibres (Fig. 5) with only occasional chronic inflammatory cells and macrophages. No zones of atypical fibroblastic proliferation were seen which might have indicated impending neoplastic change.

A different histological picture was seen in the rats from Group C. Connective tissues deep to the panniculus carnosus muscle were riddled with small cavities which had contained fragments of polyethylene and these were surrounded by a vigorous cellular response which was observed at all stages of the experiment (Fig. 6). Granulation tissue was prominent, together with siderophages, multinucleate foreign-body giant cells and chronic inflammatory elements; bone formation was a feature in some implants (Fig. 7). Despite this considerable local activity, no foci of early microscopic sarcoma (cf. Carter, 1969a, b) were identified.

Subcutaneous tissues from control rats in Groups B and D were unremarkable.

*Incidence of tumours at other sites*

The number and variety of tumours other than those found at implantation sites in the four experimental groups are listed in Table I. It will be noted that four animals developed apparently spontaneous subcutaneous sarcomas though all of them arose at some distance from the treated flank: these were a spindle cell sarcoma on the back of a control rat from Group B, a pleomorphic sarcoma on the tail of a rat in the same group, a fibromyxosarcoma in the ventral body wall of a test rat in Group C and a spindle cell sarcoma in the chest wall of a control rat in Group D. The phaeochromocytoma which was encountered in another control rat (Fig. 8) is a tumour which is rarely seen in animals of the CB strain.

*Other pathological findings*

Bronchiectasis and cystic nephritis were common in the other animals in all four groups; the incidence and severity of these changes was similar in test and control rats.

### DISCUSSION

These results confirm once again that solid pieces of polyethylene plastic induce malignant tumours when implanted into the subcutaneous tissues of rats. They also show that implants of fragmented polyethylene are only slightly less carcinogenic than the solid material and that the neoplasms produced are similar in their latent period of induction, histological appearances and behaviour. Two points in relation to the tumours may be noted. Several of them were unusually varied in appearance and contained zones of typical myxosarcoma and osteogenic sarcoma, reminiscent of the neoplasms described by Oppenheimer *et al.* (1952, 1955)—a

---

### EXPLANATION OF PLATES

FIG. 1.—Solid and shredded polyethylene prepared for implantation.

FIG. 2.—Spindle cell sarcoma associated with solid polyethylene implant. 510 days. H. and E. × 150.

FIG. 3.—Pleomorphic sarcoma associated with solid polyethylene implant. 575 days. H. and E. × 190.

FIG. 4.—Spindle cell sarcoma showing bone formation; shredded polyethylene implant. 600 days. H. and E. × 120.

FIG. 5.—Part of wall of connective tissue capsule surrounding solid plastic implant. 370 days. H. and E. × 120.

FIG. 6.—Part of local reaction in connective tissues around fragments of shredded plastic. 400 days. H. and E. × 120.

FIG. 7.—Another region of the same tissue response showing bone formation. × 120.

FIG. 8.—Phaeochromocytoma from a control rat. 650 days. Periodic acid-Schiff. × 120.

1

2

Carter and Roe.

3

4

5

6

Carter and Roe.

7

8

Carter and Roe.

diversity which presumably reflects the " intermutability of mesenchymal tissues " stressed by Willis (1967). Secondly, the incidence of metastases was low (cf. Oppenheimer, Oppenheimer and Stout, 1948; Druckrey and Schmähl, 1954; Oppenheimer *et al.*, 1955), a finding which is almost certainly due to the prompt killing of rats once a progressively growing mass was palpable in the flank. The paucity of metastases in several reports led Brunner (1959) to doubt whether some of the local tumours induced by implanted plastics were indeed malignant, but their histological appearance, proclivity for local invasion, and abnormal karyotypes (Bannerjee and Bates, 1966) should dispel such a view.

The finding that shredded polyethylene is nearly as potent a carcinogen as an intact implant of the same material is intriguing; one explanation for this may be in the different tissue responses evoked by the solid and fragmented material. It has been repeatedly shown that subcutaneous implants of solid plastics are soon enclosed in a pocket of fibrous tissue and that tumours eventually develop from elements in the walls of such pockets (Oppenheimer *et al.*, 1952, 1955, 1959; Oppenheimer, Oppenheimer, Stout, Willhite and Danishefsky, 1958). Powdered plastics are not encapsulated in this fashion (Oppenheimer *et al.*, 1959, 1961) and it is generally held that this is the main reason for their lack of carcinogenic activity. In contrast to the powders used by previous workers, the larger fragments of plastic employed in the present experiments clearly gave rise to a considerable (and sustained) local response; but instead of an organised connective tissue capsule, a more diffuse reaction was found composed mainly of granulation tissue and macrophages. Such changes are reminiscent of those produced by subcutaneous implants of plastic sponges (Dukes and Mitchley, 1962) and the shredded polyethylene may well have simulated such implants.

The size of the shredded fragments plays an important role in determining the form of the tissue response but it is not known to what extent other features of the particular polyethylene plastic may have contributed to the carcinogenic activity. The material was sterilised by irradiation before use and Carrington and Stein (1962) have shown that active free radicals may be formed in irradiated plastics. Secondly, plastics contain many added or contaminant chemicals (Scales, 1953; Little and Parkhouse, 1962) and it is possible that one of these may have contributed to the overall carcinogenic activity of the test material.

Whatever the full explanation, these anomalous results are difficult to reconcile with established theories of carcinogenesis by plastics (Bischoff and Bryson, 1964). It is almost axiomatic that fragmented plastics are not carcinogenic and that the formation of a connective tissue capsule around implants is a pre-requisite for the development of tumours. The latter view has already been questioned (Brand, Buoen and Brand, 1967) and neither axiom is borne out by the present observations. Further investigations may, however, suggest explanations which can resolve these conflicting findings.

Although this experimental study was undertaken in the light of two cases of human malignancy associated with previous extraperiosteal plombage, the full clinical implications of the findings with respect to the use of polyethylenes and other plastics in surgery are uncertain. In particular, it is not clear whether the size of the implant, relative to total body weight, influences the likelihood of tumour induction at the site of implantation. It may be argued that the local environment of an implant in terms of the number of cells with which it comes into contact varies little between species, so that the actual size of the implant is the obvious determinant, but extrapolation of results from a rat to a man on the basis of the size of the implant is extremely difficult. Furthermore, the time needed to induce cancer in a given species tends to be related to its overall life-span; an induction time of 40 to 50 weeks in a rat, which may normally live for 3 years, is equivalent to perhaps 20 to 25 years in a man. The use of plastic materials in surgery only came into use about 20 years ago, and this may be the main reason why (so far) there have been no published reports of cancer in man which could be attributed to implanted plastics.

### SUMMARY

Polyethylene plastic was implanted into the subcutaneous tissues of 40 young male rats: 20 received a solid segment of polyethylene (average weight 622 mg.) and 20 received shredded plastic (average weight 527 mg.) enclosed in a gelatin capsule. A sham operation, without implantation of plastic or capsule, was performed on 20 animals, and empty gelatin capsules were implanted into 20 control rats.

Tumours at the site of implantation occurred only in animals which received the plastic. Seven rats with solid polyethylene implants developed local tumours—one carcinoma and six sarcomas—with a latent period of induction of 48 to 79 weeks (mean 52 weeks). Five sarcomas developed in rats in association with implants of shredded plastic, their latent period of induction ranging between 41 and 77 weeks with a mean of 60 weeks. The sarcomas in the two groups were similar in appearance and behaviour. Metastases were found in two animals. The incidence of other tumours, and of non-neoplastic pathological changes, was similar in test and control rats.

Current theories of carcinogenesis by plastics are discussed in the light of these observations. The use of polyethylene in clinical medicine is also considered and the possible hazards associated with large amounts of the material, particularly in thoracic surgery, are emphasised.

We are indebted to Mr. W. P. Cleland, Professor J. G. Scadding and Dr. K. F. W. Hinson for the supply of polyethylene plastic, for information about patients under their care at the Brompton Hospital and for help in the preparation of this paper. Mr. B. C. V. Mitchley and Miss A. M. S. Walsh gave valuable technical assistance, Mr. E. Woollard provided the histology and Mr. K. G. Moreman and the staff of the photographic department prepared the photomicrographs.

This investigation was supported by grants to the Chester Beatty Research Institute from the Medical Research Council and the British Empire Cancer Campaign for Research.

### REFERENCES

BANNERJEE, M. R. AND BATES, R. R.—(1966) *Br. J. Cancer*, **20**, 555.
BISCHOFF, F. AND BRYSON, G.—(1964) *Prog. exp. Tumor Res.*, **5**, 85.
BRAND, K. G., BUOEN, L. C. AND BRAND, I.—(1967) *J. natn. Cancer Inst.*, **39**, 663.
BRUNNER, H.—(1959) *Arzneimittel-Forsch.*, **9**, 396.
CARRINGTON, A. AND STEIN, G.—(1962) *Nature, Lond.*, **193**, 976.
CARTER, R. L.—(1969a) *Br. J. Cancer*, **23**, 408.—(1969b) *J. Path.* (in press).
DRUCKREY, H. AND SCHMÄHL, D.—(1954) *Acta Un. int. Cancr.*, **10**, 119.
DUKES, C. E. AND MITCHLEY, B. C. V.—(1962) *Br. J. plast. Surg.*, **15**, 225.

HUEPER, W. C. AND CONWAY, W. D.—(1964) 'Chemical carcinogenesis and cancer'. Springfield, Illinois (Thomas, Ltd.).
LITTLE, K. AND PARKHOUSE, J. (1962) *Lancet*, ii, 857.
LUCAS, B. G. B. AND CLELAND, W. P.—(1950) *Thorax*, **5**, 248.
NOTHDURFT, H.—(1955) *Naturwissenschaften*, **42**, 106.
OPPENHEIMER, B. S., OPPENHEIMER, E. T., DANISHEFSKY, I., STOUT, A. P. AND EIRICH, F. R.—(1955) *Cancer Res.*, **15**, 333.
OPPENHEIMER, B. S., OPPENHEIMER, E. T. AND STOUT, A. P.—(1948) *Proc. Soc. exp. Biol. Med.*, **67**, 33.—(1952) *Proc. Soc. exp. Biol. Med.*, **79**, 366.
OPPENHEIMER, B. S., OPPENHEIMER, E. T., STOUT, A. P., DANISHEFSKY, I. AND WILLHITE, M.—(1959) *Acta Un. int. Cancr.*, **15**, 659.
OPPENHEIMER, B. S., OPPENHEIMER, E. T., STOUT, A. P., WILLHITE, M. AND DANISHEFSKY, I.—(1958) *Cancer, N.Y.*, **11**, 204.
OPPENHEIMER, E. T., WILLHITE, M., DANISHEFSKY, I. AND STOUT, A. P.—(1961) *Cancer Res.*, **21**, 132.
SCALES, J. T.—(1953) *Proc. R. Soc. Med.*, **46**, 647.
WILLIS, R. A.—(1967) 'Pathology of tumours'. 4th Edition. London (Butterworths).